# Path Following for Autonomous Mobile Robots with Deep Reinforcement Learning

**DOI:** 10.3390/s24020561

**Published:** 2024-01-16

**Authors:** Yu Cao, Kan Ni, Takahiro Kawaguchi, Seiji Hashimoto

**Affiliations:** Program of Intelligence and Control, Cluster of Electronics and Mechanical Engineering, School of Science and Technology, Gunma University, 1-5-1 Tenjin-cho, Kiryu 376-8515, Japan; t202d602@gunma-u.ac.jp (Y.C.); t202d003@gunma-u.ac.jp (K.N.); kawaguchi@gunma-u.ac.jp (T.K.)

**Keywords:** autonomous mobile robot, path following, velocity control, deep reinforcement learning, soft actor-critic

## Abstract

Autonomous mobile robots have become integral to daily life, providing crucial services across diverse domains. This paper focuses on path following, a fundamental technology and critical element in achieving autonomous mobility. Existing methods predominantly address tracking through steering control, neglecting velocity control or relying on path-specific reference velocities, thereby constraining their generality. In this paper, we propose a novel approach that integrates the conventional pure pursuit algorithm with deep reinforcement learning for a nonholonomic mobile robot. Our methodology employs pure pursuit for steering control and utilizes the soft actor-critic algorithm to train a velocity control strategy within randomly generated path environments. Through simulation and experimental validation, our approach exhibits notable advancements in path convergence and adaptive velocity adjustments to accommodate paths with varying curvatures. Furthermore, this method holds the potential for broader applicability to vehicles adhering to nonholonomic constraints beyond the specific model examined in this paper. In summary, our study contributes to the progression of autonomous mobility by harmonizing conventional algorithms with cutting-edge deep reinforcement learning techniques, enhancing the robustness of path following.

## 1. Introduction

Autonomous Mobile Robots (AMRs) refer to robotic systems designed to exhibit minimal or no human intervention in their movement [1]. These robots are engineered to autonomously follow a predefined path, whether in indoor or outdoor environments. AMRs are increasingly appreciated for their expanding applications across diverse domains, including logistics transportation [2], security surveillance [3], and robotic cleaning services [4]. Within the customer service industry, an escalating number of AMRs are being deployed to enhance customer experiences by providing daily life conveniences [5,6]. Moreover, the agricultural industry is increasingly expressing interest in AMRs, driven by their ability to address issues such as labor shortages, natural phenomena, and economic challenges that have the potential to significantly diminish opportunities in farming [7].

The challenge of following a predetermined path has long been a focal point in the control engineering community. Path following involves a vehicle navigating a globally defined geometric path with loose time constraints and can be divided into control theory-based methods and geometric methods [8]. Control theory-based methods, such as Proportional–Integral–Derivative (PID) controllers, face challenges in finding optimal parameters [9]. Fuzzy controllers rely on expert experience or prior knowledge [10], while model predictive controllers require consideration of computational costs and precise modeling for reliable results [11]. In comparison to control theory-based methods, geometric methods have become more popular due to their simplicity, robustness, and suitability for real-time control. The Pure Pursuit (PP) controller, proposed as the earliest geometric approach for path following, fits a circle through the vehicle’s current position to a point on the path ahead of the vehicle by a look-ahead distance [8,12]. It was first discussed in [13] and later formally elaborated in [14], where the PP strategy and its applications were introduced. The straightforward nature of this strategy has contributed to its popularity in various applications. Notably, the PP controller has been employed in two vehicles during the DARPA Grand Challenge [15] and three vehicles in the DARPA Urban Challenge [16]. However, this approach assumes that the vehicle is operating at a constant speed and the path is free of curvature, leading to degraded performance on curved paths [12,17]. Additionally, especially when the vehicle deviates from the path, and the distance between the vehicle and the path exceeds the look-ahead distance, there is no corresponding control law.

In recent years, Reinforcement Learning (RL) has achieved remarkable success in various fields, particularly in robotics, garnering increased attention and widespread recognition [18]. RL is a machine learning method that addresses the challenge of enabling a decision-making agent to learn optimal actions within an environment. The introduction of Deep Neural Networks (DNNs) into RL, owing to their outstanding ability to approximate nonlinear functions and extract relevant features from raw inputs, has given rise to the advent of Deep Reinforcement Learning (DRL). This approach excels in tasks such as defeating the world champion in the game of Go [19] and mastering intricate robotics manipulation tasks [20]. Naturally, DRL has found applications in path following. Liu et al. [21] introduced a multiple kernel feature learning framework for value function approximation, addressing the challenges of feature representation and online learning ability in RL. Their simulation results demonstrated better performance in tracking precision and smoothness. Chen et al. [22] proposed a steering control approach that combines PID control and PP control. In this setup, RL is employed to learn the weights of the two controllers, balancing the trade-off between smooth control and tracking error. Subsequently, they extended their work by updating the constant speed control to a new speed adaptation method using fuzzy logic [23]. This modification allows the original approach to be applicable not only to low-speed urban environments, but also to high-speed scenarios, reaching speeds of up to 80 km/h. Chen et al. [24] presented a hybrid approach combining DRL and PP control. Similarly, the steering output is a combination of the outputs from both, treating DRL as a compensatory mechanism for PP control. The previously mentioned methods primarily emphasize steering control, with many adopting constant speed control, which lacks generality. Moreover, in certain instances, manual design of reference speeds is adopted, demanding additional optimization efforts.

In this paper, our focus is on exploring the combination of PP control and DRL to address the challenges in path following. PP is responsible for steering control, while DRL, specifically employing the Soft Actor-Critic (SAC) algorithm, takes charge of velocity control, creating a complementary relationship between the two. We believe that the implementation of PP, being an easily deployable method, can compensate for the shortcomings of the algorithm itself through adaptive velocity adjustment, resulting in improved path convergence. Simulation and experimental results, validated using a nonholonomic mobile robot, demonstrate the ease of training for our proposed approach and its superiority in tracking paths with varying curvatures.

## 2. Problem Formulation

In this section, we introduce the problem of path following for nonholonomic mobile robots in a planar environment.

### 2.1. Kinematics Modeling

**Figure 1:**
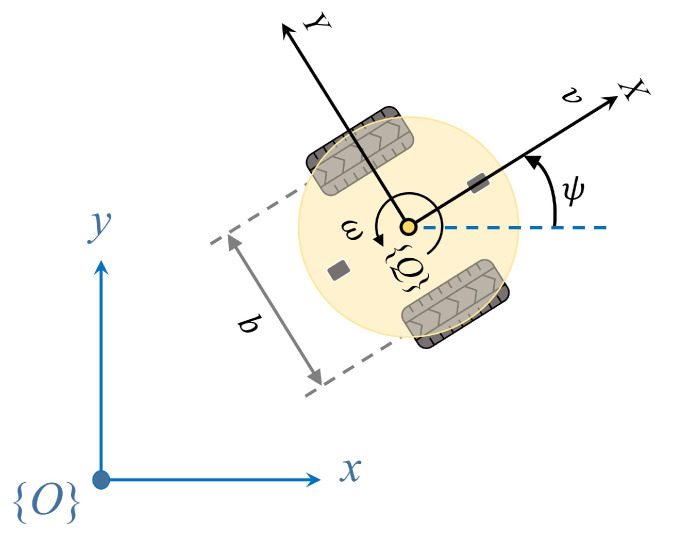
A two-wheeled independently driven nonholonomic mobile robot with the definition of the global coordinate frame {O} and the body coordinate frame {Q} .

Nonholonomic mobile robots constitute a category of mobile robots with constrained mobility, unlike typical holonomic mobile robots that can move freely in a plane. Steering in nonholonomic mobile robots is accomplished by independently controlling the speed of the wheels on each side of the vehicle. When the speeds of the wheels on both sides are not equal, the vehicle will turn [25]. Basic differential steering robots are equipped with two driven wheels and a front and rear caster for added stability, as illustrated in Figure 1. In the context of a mobile robot situated on a 2D plane with a defined global Cartesian coordinate system {O} , the robot possesses three degrees of freedom represented by its posture,

(1) $$x=\left[\begin{array}{c} x\\ y\\ \psi \end{array}\right]$$

where (x,y) represents the robot’s current position in the global coordinate system, and ψ represents the heading angle, measured counterclockwise from the *x*-axis.

The mobile robot’s motion is controlled by its linear velocity *v* and rotational velocity ω , as their positive directions are defined in Figure 1. The mobile robot’s kinematics is then defined as follows [25,26,27]:

(2) $$\dot{x}=\left[\begin{array}{c} \dot{x}\\ \dot{y}\\ \dot{\psi } \end{array}\right]=\left[\begin{array}{cc} \cos\psi & 0\\ \sin\psi & 0\\ 0 & 1 \end{array}\right]u$$

where $u={\left[v,\omega \right]}^{T}\in U\subset R^{2}$ define input constraints. Path following of such a nonholonomic wheeled mobile robot involves the design of algorithms to generate reference commands for u .

Let $v_{max}$ represent the maximum linear velocity achievable by the mobile robot. It is crucial to account for the condition that, even when the robot simultaneously moves forward at a linear velocity *v* and rotates at an angular velocity ω , the velocity of the outer wheel should not exceed the maximum allowed velocity [27]. Therefore, the following constraint is applied:

(3) $$\hat{v}+\frac{b}{2}\hat{\omega }\leq v_{max}$$

where $\hat{v}$ and $\hat{\omega }$ represent the set maximum velocities in practical use, and *b* denotes the wheelbase, which is the distance between the centers of the wheels. The experimental robot in the paper is tested to move at a maximum velocity slightly exceeding 0.5 m/s. Through the constraint outlined in Equation (3), the maximum values are determined and presented in Table 1.

### 2.2. Path Following

The objective of path following is to design a controller, such that the mobile robot follows an arc-length parametrized reference path [11],

(4) $$P=\{p\in R^{2}|p=p_{r}\left(\lambda \right),\forall \lambda \geq 0\}.$$

For any given parameter λ , a local reference coordinate system {R} centered at $p_{r}\left(\lambda \right)$ can be defined, denoted by the subscript *r*. The relative angle $\delta_{r}$ between the global coordinate system {O} and the local reference coordinate system {R} can be calculated with Equation (5).

(5) $$\delta_{r}\left(\lambda \right)=atan2\left(y_{r}^{\prime }\left(\lambda \right),x_{r}^{\prime }\left(\lambda \right)\right)$$

where the function atan2 used here is the four-quadrant version of arctan, which calculates the angle between the positive *x*-axis and the robot position ${\left[x_{r},y_{r}\right]}^{T}$ in the Cartesian plane, with a positive counterclockwise direction. $x_{r}^{\prime }$ and $y_{r}^{\prime }$ are the first order derivatives. Moreover, it is clear that the parametrized reference path must exhibit continuous differentiability.

Considering the robot’s posture at time t as [x(t),y(t),ψ(t)] , the error in path following, commonly referred to as the cross-track error, is determined by Equation (6), which is a cross product between two vectors [12]. The control objective is to guarantee that the cross-track error converges such that $\lim_{t\to \infty }e_{p}\left(t\right)=0$ .

(6) $$e_{p}\left(t\right)=d_{y}{\hat{t}}_{x}-d_{x}{\hat{t}}_{y}$$

where $d=(d_{x},d_{y})$ is the tracking error vector and $\hat{t}=\left({\hat{t}}_{x},{\hat{t}}_{y}\right)$ is the unit tangent vector to the reference path at λ(t) , as defined in Equations (7) and (8), respectively.

(7) $$d=\left(x\left(t\right),y\left(t\right)\right)-(x_{r}\left(\lambda \left(t\right)\right),y_{r}\left(\lambda \left(t\right)\right))$$

(8) $$\hat{t}=\frac{\left(x_{r}^{\prime }\left(\lambda \left(t\right)\right),y_{r}^{\prime }\left(\lambda \left(t\right)\right)\right)}{∥\left(x_{r}^{\prime }\left(\lambda \left(t\right)\right),y_{r}^{\prime }\left(\lambda \left(t\right)\right)\right)∥}$$

**Figure 2:**
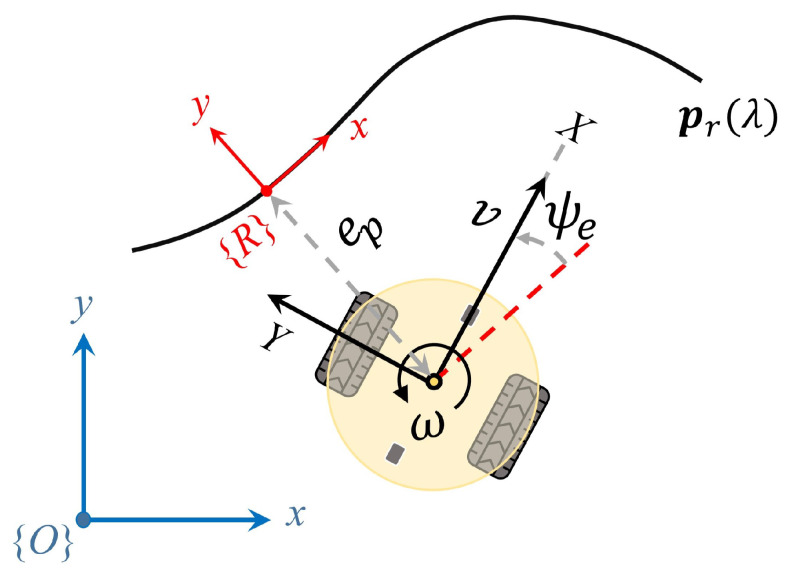
Schematic representation of cross-track error $e_{p}$ and heading error $\psi_{e}$ with respect to the reference path $p_{r}\left(\lambda \right)$ .

The orientation error $\psi_{e}\left(t\right)$ between the robot and the reference path at time *t* is determined by Equation (9), which is frequently incorporated and is typically treated as a secondary objective, or used to assist in the elimination of the cross-track error. It indicates moving towards or away from the direction of the path.

(9) $$\begin{array}{cc} \psi_{e}\left(t\right) & =\psi \left(t\right)-\delta_{r}\left(\lambda \left(t\right)\right)\\ \psi_{e}\left(t\right) & =atan2(\sin\left(\psi_{e}\left(t\right)\right),\cos\left(\psi_{e}\left(t\right)\right)) \end{array}$$

where $\psi_{e}\left(t\right)$ is normalized within the range of $\left[-\pi ,\pi \right]$ . While the trigonometric operations remain unchanged, constraining the range to this specific interval aids in reducing the observation space. A graphic representation of the path following errors is illustrated in Figure 2.

To determine the point along the reference path for calculating the cross-track error, the point nearest to the robot is selected [12,28]. It leads to an optimization problem of finding the parameter λ that minimizes the distance between the robot’s position and the reference path. The optimization problem can be expressed as in Equation (10), with a preference for the squared Euclidean distance due to its equivalence with the original optimization problem and computational convenience.

(10) $$\lambda \left(t\right)=\underset{\lambda }{\arg\min}{∥\left(x\left(t\right),y\left(t\right)\right)-\left(x_{r}\left(\lambda \right),y_{r}\left(\lambda \right)\right)∥}^{2}$$

A common approach for updating the path variable λ involves iteratively computing the value that minimizes the distance between the robot and the reference path. This process can be accomplished through the application of the conjugate gradient method, which is highly efficient when solving quadratic convex optimization problems, often converging to the optimal solution within a finite number of steps [29]. Furthermore, the feature of guaranteeing only a local optimum serves to prevent abrupt jumps in the path parameter, ensuring stability in the optimization process. Further implementation details can be found in [29,30].

**Figure 3:**
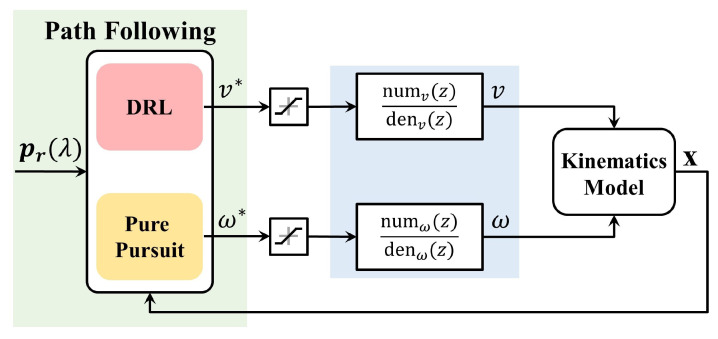
Separate longitudinal and latitudinal control structure of the proposed path-following control system.

In the end, the proposed path-following control system is illustrated in Figure 3. In this system, the path following algorithm utilizes both path information and model states as inputs to compute errors. Subsequently, the commands for linear velocity and angular velocity are generated by the DRL and PP, respectively. The actual current velocities undergo saturation processing and are related through transfer functions as inputs to the kinematics model. Here, we consider the relationship between the velocity command and velocity as an identity transform to simplify the upcoming analysis. This consideration is also made to fulfill the Markov property, where the future evolution of a process depends solely on the present state and not on past history.

## 3. Design and Implementation

In this section, we introduce our proposal for path following based on DRL. Firstly, we present a variant of PP utilized in steering control. The aim of this variant is to better align with our proposal and address an inherent issue in PP. Following that, we delve into the application of SAC and the design of a training environment with the objective of minimizing cross-track error while encouraging the maximization of linear velocity. Finally, we discuss implementation details.

### 3.1. Pure Pursuit Steering Control

The original pure pursuit algorithm fits a circle between the robot’s position and a look-ahead point on the reference path, assuming that the robot moves along this trajectory. The conventional selection for the look-ahead point is a point on the reference path such that $∥\left(x,y\right)-(x_{r}\left(\lambda \right),y_{r}\left(\lambda \right))∥=L$ , representing a distance *L* from the robot’s current position. Since there are potentially multiple points fulfilling this criterion, the one with the highest value of the parameter λ is selected. The main issue with this selection method is that when the robot deviates from the path by more than the distance *L*, the control law is not defined [12]. Consequently, the failure to stay within the distance *L* results in the optimization problem’s failure.

**Figure 4:**
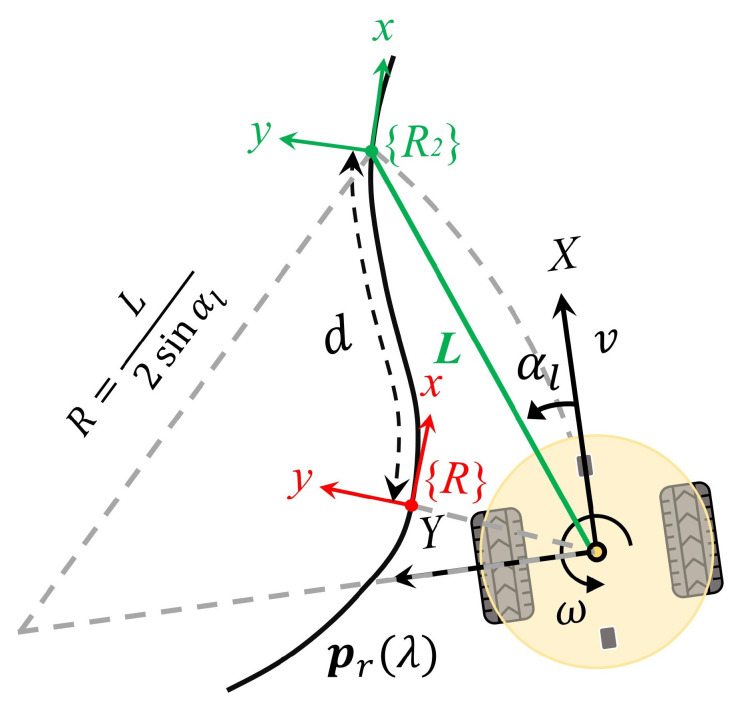
Geometry of the variant pure pursuit algorithm, which ensures defined control at arbitrary positions.

In this paper, a variant is proposed with the primary objective of addressing the aforementioned issues. Leveraging an arc-length parameterized path, an enhancement can be achieved by choosing a point situated at an arc length of *d* forward along the reference path from the point closest to the robot’s current position relative to the path, denoted as $\left[x_{r}\left(\lambda \right),y_{r}\left(\lambda \right)\right]$ . This newly selected point, denoted as $\left[x_{r}\left(\lambda +d\right),y_{r}\left(\lambda +d\right)\right]$ , is then assigned as the look-ahead point, as illustrated in Figure 4. The look-ahead distance is subsequently calculated as $L=∥\left(x,y\right)-(x_{r}\left(\lambda +d\right),y_{r}\left(\lambda +d\right))∥$ . Another advantage accompanying such a choice is that we only need to solve the optimization problem once, specifically for the nearest point.

Eventually, the commanded heading rate $\omega^{*}$ for a robot traveling at linear velocity *v* is defined as per Equation (11).

(11) $$\omega^{*}=\frac{2v\sin\alpha_{l}}{L}$$

where the look-ahead angle $\alpha_{l}$ is given by

(12) $$\alpha_{l}=\arctan\left(\frac{y_{r}\left(\lambda +d\right)-y}{x_{r}\left(\lambda +d\right)-x}\right)-\psi .$$

### 3.2. Soft Actor-Critic in Velocity Control

A Markov Decision Process (MDP) is characterized by a sequential decision process that is fully observable, operates in a stochastic environment, and possesses a transition model adhering to the Markov property [31]. The MDP can be concisely represented as a tuple (S,A,p,r) , where S represents the set of all states called the state space; A denotes the actions available to the agent called the action space; p:S×S×A→[0,1] denotes the transition probability $p(s^{\prime }|s,a)$ , representing the likelihood that action a in state s will result in state $s^{\prime }$ ; and r:S×A represents the immediate reward received after transitioning from state s to state $s^{\prime }$ as a result of action a .

Detailed knowledge and the algorithm of SAC can be referenced from [32,33]. Here, we introduce only the essential components used in the proposed system. SAC is a RL algorithm built upon the Maximum Entropy RL (MERL) framework, which generalizes the objective of standard RL by introducing a regularization term. This regularization term ensures that the optimal policy $\pi^{*}$ maximizes both expected return and entropy simultaneously as follows:

(13) $$\pi^{*}=\underset{\pi }{\arg\max}\sum_{t}E_{\left(s_{t},\;a_{t}\right)∼\rho^{\pi }}\;\left[r\left(s_{t},a_{t}\right)+\alpha H\left(\pi \left(\cdot |s_{t}\right)\right)\right]$$

where $\rho^{\pi }$ represents the trajectory of state-action pairs that the agent encounters under the control policy π , and $H(\pi \left(\cdot |s_{t}\right))$ is the entropy associated with the parameter α . This parameter acts as the temperature, influencing the balance between the entropy term and the reward.

The soft Q-function is formulated to evaluate state-action pairs, as outlined in Equation (14).

(14) $$Q\left(s_{t},a_{t}\right)=r\left(s_{t},a_{t}\right)+\gamma E_{s_{t+1}∼p}\left[V\left(s_{t+1}\right)\right]$$

where γ represents the discount rate for preventing the infinitely large return, and the soft state-value function $V(s_{t})$ based on MERL is defined by

(15) $$V\left(s_{t}\right)=E_{a_{t}∼\pi }\left[Q\left(s_{t},a_{t}\right)-\alpha \log\pi \left(a_{t}|s_{t}\right)\right]$$

The optimization is performed for function approximators of both the soft Q-function and the policy. The soft Q-function is parameterized by $\theta \in R^{n}$ , representing a vector of *n* parameters, and can be effectively modeled using a DNN. The optimization of the soft Q-function is achieved by employing a policy evaluation algorithm, such as Temporal-Difference (TD) learning. The parameters of the soft Q-function can be optimized by minimizing the mean squared loss given by Equation (16). The loss is approximated using state-action pairs stored in the experience replay buffer, denoted by D .

(16) $$L_{Q}\left(\theta \right)=E_{s_{t},a_{t}∼D}\left[{\left(Q_{\theta }\left(s_{t},a_{t}\right)-y_{t}\right)}^{2}\right]$$

where $y_{t}$ given in Equation (17) is the TD target that the soft Q-function is updating towards. The update makes use of a target soft Q-function with parameters $\bar{\theta }$ , where i=1,2 denotes the double Q-function approximators, referred to as the clipped double-Q trick [34].

(17) $$y_{t}=r\left(s_{t},a_{t}\right)+\gamma E_{s_{t+1}∼p}\left[\min_{i=1,2}Q_{\bar{\theta_{i}}}\left(s_{t+1},a_{t+1}\right)-\alpha \log\pi \left(a_{t+1}|s_{t+1}\right)\right]$$

Similarly, the policy, parameterized by $\phi \in R^{m}$ , is modeled as a Gaussian with a mean and a standard deviation determined by a DNN. The objective for updating the policy parameters is defined by maximizing the expected return and the entropy, as depicted in Equation (18). Due to the challenges in directly sampling latent action and computing gradients, the policy is reparameterized such that $a_{t}=f_{\phi }\left(\xi_{t};s_{t}\right)=\tanh\left(\mu_{\phi }\left(s_{t}\right)+\sigma_{\phi }\left(s_{t}\right)⊙\xi_{t}\right)$ within a finite bound. Here, $\mu_{\phi }$ and $\sigma_{\phi }$ represent the mean and standard deviation of the action, respectively, and $\xi_{t}∼N\left(0,I\right)$ is an input noise vector. The reparameterized sample is thus differentiable.

(18) $$J_{\pi }\left(\phi \right)=E_{s_{t}∼D,\;\xi_{t}∼N}\left[\min_{i=1,2}Q_{\theta_{i}}\left(s_{t},f_{\phi }\left(\xi_{t};s_{t}\right)\right)-\alpha \log\pi_{\phi }\left(f_{\phi }\left(\xi_{t};s_{t}\right)|s_{t}\right)\right]$$

where $\pi_{\phi }$ is implicitly defined in relation to $f_{\phi }$ .

In practice, the temperature α is adjusted automatically to constrain the average entropy of the policy, allowing for variability in entropy at different states. The objective of discovering a stochastic policy with maximal expected return, satisfying a minimum expected entropy constraint, is presented in Equation (19).

(19) $$J\left(\alpha \right)=E_{a_{t}∼\pi_{t}}\left[-\alpha \log\pi_{t}\left(a_{t}|s_{t}\right)-\alpha \bar{H}\right]$$

where the entropy target $\bar{H}$ is set to be the negative of the action space dimension.

**Figure 5:**
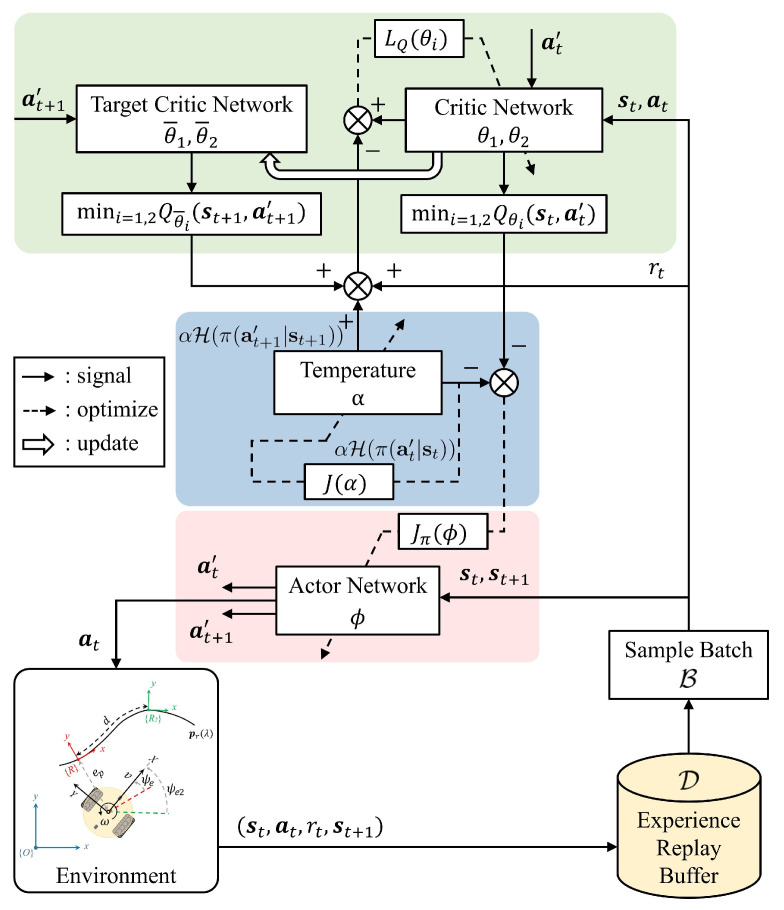
The optimization process of SAC-based controller at each time-step. In order to explicitly distinguish between different sources, $a^{\prime }$ represents an action that is resampled from the current policy, rather than being drawn from prior experiences.

The overall architecture of the SAC-based controller is depicted in Figure 5. It employs a pair of neural networks—one specifically designed for learning the policy and the other for learning the value function. To mitigate bias in value estimation, target networks are introduced. Following the optimization of the networks, the parameters of the target networks undergo an update using a soft update strategy, denoted by Equation (20). Specifically, a fraction of the updated network parameters is blended with the target network parameters.

(20) $$\bar{\theta }\leftarrow \tau \theta +\left(1-\tau \right)\bar{\theta }$$

where parameter τ indicates how fast the update is carried on and the update is performed at each step after optimizing the online critic networks.

Moreover, an experience replay buffer D for storing and replaying samples, effectively reducing sample correlation, enhancing sample efficiency, and improving the learning capability of the algorithm is integrated. Through interaction with the training environment, the mobile robot, acting as the agent, takes an action $a_{t}$ based on the current policy according to observed states $s_{t}$ , receives immediate rewards $r_{t}$ , and transitions to the next state $s_{t+1}$ . This experience is stored in the replay buffer. During each optimization step, a mini-batch sample B is randomly drawn from the buffer to approximate the required expected values. The detailed description of the designed training environment is provided below.

#### 3.2.1. Observation Space and Action Space

The observation s , which serves as the input to the velocity controller for path following, is designed as follows:

(21) $$s=\{e_{p},\psi_{e},v,\omega ,\psi_{e2}\}$$

where $e_{p}$ is the cross-track error, $\psi_{e}\in \left[-\pi ,\pi \right]$ is the normalized orientation error between the path and the mobile robot, *v* and ω are the current linear velocity and rotational velocity of the robot, respectively. $\psi_{e2}$ , selected from the look-ahead point as discussed in the previous section, functions as an augmented observation that provides information about the curvature of the path in the future.

**Figure 6:**
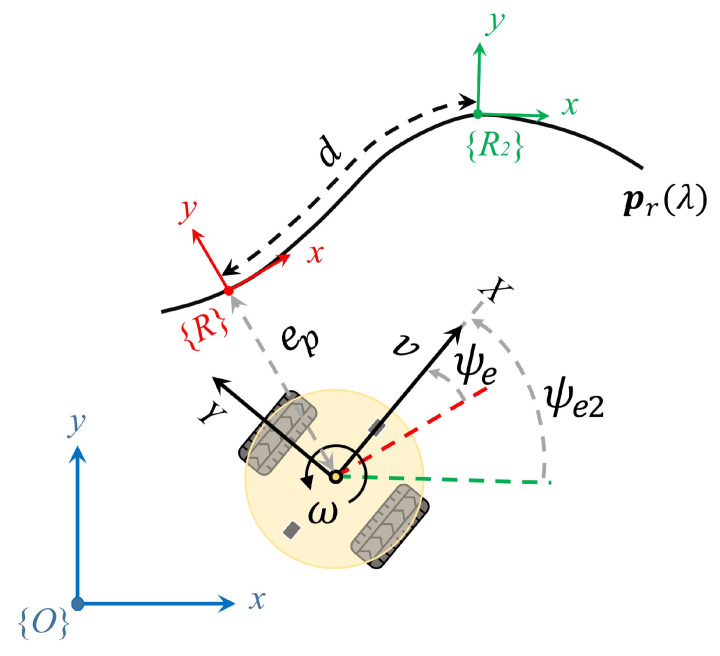
Observations concerning a predetermined reference path.

A graphical explanation is presented in Figure 6. Through trial and adjustment, the arc-length divergence parameter *d*, which serves both steering control and the observations in this paper, is set to 0.2 m. In configuring this parameter, our primary consideration is on selecting a look-ahead point that ensures the robot quickly regains the path. A small value of *d* makes the robot approach the path rapidly, but it may result in overshooting and oscillations along the reference path. Conversely, a large *d* reduces oscillations but might increase cross-track errors, especially around corners.

The action, denoted as a(t)=π(s(t)|ϕ) , represents the rate of linear velocity $\dot{v}$ normalized by the velocity itself. This selection is adopted to mitigate undesired rapid changes. Adopting an incremental control input for the robot makes it easier to achieve smooth motions without the need for additional rewards or penalties for excessive velocity changes. Furthermore, constraining the velocity rate within a specified range, i.e., $\dot{v}\in \left[{\dot{v}}_{min},{\dot{v}}_{max}\right]$ , provides a better stability. The linear velocity command $v^{*}\left(t\right)$ after saturation operation for the mobile robot at each time step is calculated using Equation (22).

(22) $$\begin{array}{cc} a(t) & =\tanh(\mu_{\phi }\left(s\left(t\right)\right)+\sigma_{\phi }\left(s\left(t\right)\right)⊙\xi \left(t\right))\\ {\dot{v}}^{*}\left(t\right) & =ka(t)+b\\ v^{*}\left(t\right) & =\mathrm{clip}(v\left(t\right)+{\dot{v}}^{*}\left(t\right)\Delta t,v_{min},v_{max}) \end{array}$$

where $k=({\dot{v}}_{max}-{\dot{v}}_{min})/2$ and $b=({\dot{v}}_{max}+{\dot{v}}_{min})/2$ are the scale and bias, respectively, to recover the normalized action to the range of the desired action. The range of linear velocity rate is designed as [−0.5,0.3] m/s^2^. The exploration space for deceleration is slightly larger than that for acceleration, addressing situations requiring urgent braking.

#### 3.2.2. Reward Function

The reward function is designed to penalize the robot when it deviates from the path, while rewarding the robot’s velocity as much as possible, as depicted in Equation (23).

(23) $$r\left(t\right)=-k_{1}\left|e_{p}\left(t\right)\right|+k_{2}v\left(t\right)\left(1-\frac{1}{e_{tol}}\left|e_{p}\left(t\right)\right|\right)-k_{3}F\left(t\right)$$

where $k_{1}$ , $k_{2}$ , and $k_{3}$ are positive constants that define the importance of each term. $e_{tol}$ is the tolerance for cross-track error within which the robot receives positive velocity rewards. Based on intuitive considerations, it is desirable for the robot to decrease its velocity when deviating from the path to prevent further error expansion. To achieve this, a segmented penalty approach is also introduced. When the cross-track error exceeds a critical threshold, the penalty on velocity increases accordingly. This design ensures that the policy receives velocity rewards only when the cross-track error is within the critical threshold.

**Figure 7:**
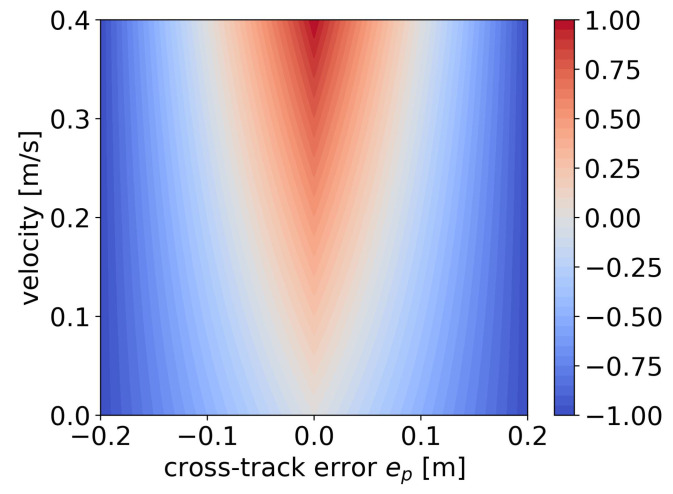
Reward function for path following within the $e_{p}$ range of [−0.2,0.2] m.

The reward function with the first two terms is visualized in Figure 7, with the range of $\left[-e_{tol},e_{tol}\right]$ . Within this range, the reward at each step ranges from a maximum value of 1, indicating perfect tracking of the path at maximum speed, to a minimum value of −1 . Due to improvements in the pure pursuit algorithm, the mobile robot can consistently track the point ahead of itself on the path at any lateral distance. In this scenario, it is sufficient to solely investigate the reward associated with the robot traveling along the path.

Additionally, it has been observed in experiments that the agent may choose to discontinue forward movement at challenging turns to avoid potential penalties. To address this situation, a flag *F* defined as

$$F\left(t\right)=\left\{\begin{array}{cc} 1 & \mathrm{if}\;v(t)<\epsilon ,\\ 0 & \mathrm{otherwise}. \end{array}\right.$$

indicating a stationary state has been introduced, where ϵ is an extremely small value such as $1\times 10^{-6}$ for numerical stability. The parameters for the reward function are designed as follows: $k_{1}=5.0$ , $k_{2}=2.5$ , $k_{3}=0.2$ , and $e_{tol}=0.2$ m.

#### 3.2.3. Environment and Details

**Figure 8:**
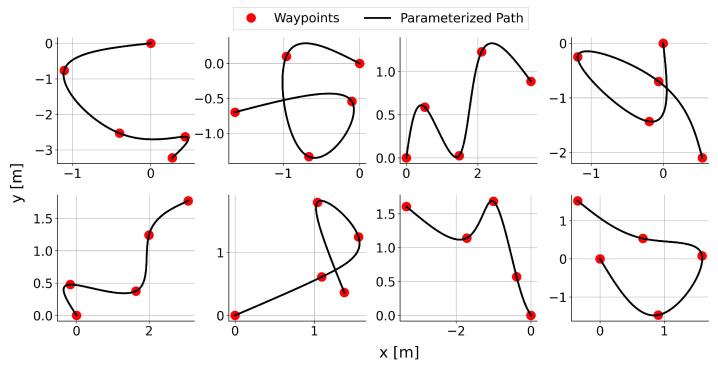
Example of randomly generated reference paths.

The training environment encompasses the kinematics of the robot itself and a reference path. Ensuring the adaptability of the policy to various challenges is crucial, as it cultivates the ability to handle generalized scenarios, thereby reducing the risk of overfitting to specific paths. We employed a stochastic path generation algorithm proposed in our previous work [35], randomly generating a reference path for the robot to follow at the beginning of each episode. In this paper, the parameters of the stochastic path generation algorithm is defined with $N_{w}=5$ , $L_{min}=0.5$ m, and $L_{max}=2.0$ m. Straight paths with a generation probability of 0.1 are also introduced into the training. The straight path is achieved by setting $N_{w}=2$ and $L_{w}=2.5$ m. The example of randomly generated curved paths is illustrated in Figure 8.

After the generation of a reference path, the initial posture of the robot is randomly sampled from a uniform distribution, with a position error range of [−0.1,0.1] meters for both the global *x*-axis and *y*-axis, and a heading error range of [−0.0873,0.08723] radians with respect to the reference path. A warm-up strategy is also implemented to gather completely random experiences. During the initial training phase, the agent takes random actions uniformly sampled from the action space. Each episode terminates when the robot reaches the endpoint, or when reaching 400 time steps. The summarized training parameters are presented in Table 2.

The actor and critic neural networks are both structured with two hidden layers. Each layer is equipped with Rectified Linear Unit (ReLU) activation function, featuring 256 neurons in both hidden layers. The actor’s final layer outputs the mean $\mu_{\phi }\left(s_{t}\right)$ and standard deviation $\sigma_{\phi }\left(s_{t}\right)$ of a distribution, facilitating the sampling of a valid action. Subsequently, the action undergoes a tanh transformation to confine its range, as outlined in Equation (22). In the critic network, the action and state are concatenated to form an input. For the optimization of neural networks, the Adam optimizer [36] is utilized with a minibatch size of 256. Subsequently, training is conducted five times separately, allowing for an assessment of the algorithm’s effectiveness and stability. Each training utilizes a distinct random seed to control factors such as path generation parameters and the initial posture of the robot. This approach ensures the reproducibility of the experiments. Hyperparameters are summarized in Table 3.

## 4. Results and Analysis

In this section, we discuss the achievements attained by the path following control of the proposed method. We first analyze the training process and then introduce two evaluation criteria, failure rate and completion rate, to assess the advantages of the proposed method over PP control in the majority of samples. Finally, we test and analyze the superiority of the proposed method over PP control in both simulation and experimental environments.

### 4.1. Training Process

**Figure 9:**
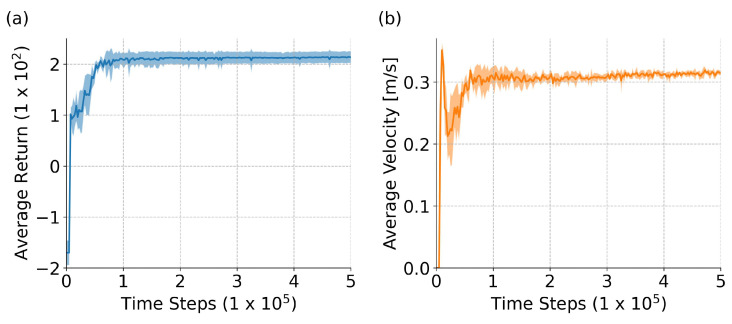
Learning curves for path following over 5 trials: (**a**) average return; (**b**) average velocity. The solid line represents the mean, while the shaded area corresponds to the confidence interval represented by the standard deviation.

The learning curves of average return and average velocity for the path following problem are depicted in Figure 9. In the initial stage, where the average velocity consistently increases, the learned policy exhibits a relatively high average velocity but with lower rewards obtained. This suggests that the learned policy prioritizes speed improvement while neglecting the reduction in the cross-tracking error. Additionally, the initial stage of the five trials demonstrates a remarkably high level of consistency, with minimal standard deviation (noted by the absence of shaded regions in the figures). Subsequently, the average velocity noticeably decreases, while rewards, on the contrary, increase. This indicates that the policy starts learning how to decelerate to handle curves, especially challenging bends, resulting in a certain degree of over-deceleration.

After progressing halfway through the learning process, a more suitable policy is identified that balances both velocity and cross-track error. For all five trials, the overall training tends to stabilize. Moreover, compared to other OpenAI Gym RL tasks [37] that often require steps in the order of tens of millions [32,33], our approach converges with fewer steps, confirming the ease and stability of algorithm convergence.

### 4.2. Simulation Results

#### 4.2.1. Quantitative Evaluation

The performance of both the PP control and the proposed method is assessed across 1000 paths generated using the same algorithm employed during training. Performance is evaluated by checking whether the cross-track error surpasses a predefined threshold within a specified number of time steps, set at 400 steps, aligning with the duration used for training the SAC-based controller per episode. In each path-following task, if this threshold is exceeded, the task is marked as a failure, and the execution of the task is terminated. The failure rate is then defined as the proportion of failures out of the total 1000 tasks.

(24) $$\mathrm{failure}\;\mathrm{rate}=\frac{N_{failure}\;\left[\mathrm{sample}\right]}{N_{total}\;\left[\mathrm{sample}\right]}$$

where $N_{failure}$ represents the number of failed samples. Additionally, the overall completion of the path is evaluated at that specific moment.

With the path parameterized by arc length, the completion rate is defined as the ratio of the path parameter at which the robot concludes the task to the parameter of the path’s endpoint, as expressed in Equation (25). This parameterization allows for a meaningful measure of how much of the path has been covered when the robot finishes its trajectory.

(25) $$\mathrm{completion}\;\mathrm{rate}=\frac{\lambda_{n}\;\left[m\right]}{\lambda_{end}\;\left[m\right]}$$

where $\lambda_{n}$ denotes the arc length parameter of the nearest point as in Equation (10), while $\lambda_{end}$ represents the arc length parameter of the path’s endpoint.

Firstly, the performance of the PP control is evaluated. The results of failure rate and completion rate for three different thresholds of cross-track error (0.1, 0.2, and 0.3 m) are summarized in Table 4 and Table 5, respectively. From the perspective of failure rate, it is certain that a higher threshold leads to a lower failure rate for any given velocity. Within the same threshold criteria, as reference velocity increases, the failure rate also increases, primarily due to poor performance at high velocities in turns. Conversely, from the perspective of completion rate, lower velocities may result in minimal failure in path following, but the overall completion rate is not high. Increasing velocity is associated with an improvement in completion rate, but beyond a certain velocity, the completion rate decreases due to premature failures caused by excessive velocity. Moreover, the higher the velocity, the greater the variation in completion rates across different paths.

Secondly, the five trained policies are evaluated on the same set of 1000 paths. The trained policies exhibit significant improvements in both the failure rate and completion rate. At the most stringent threshold of 0.1 m, there is a low failure rate. Despite these failures, the completion rate reaches as high as 0.873, representing the average result of the five policies. Beyond the 0.2 m threshold, the absence of failures and the path completion rate approaching 1 imply that, after training the velocity control ensures a reduction in cross-track error while maximizing velocity in regions with low curvature. In other words, it allows for higher velocities when possible and slows down where necessary. The reason for not reaching 1 is that some randomly generated paths have a substantial arc length, and they cannot be fully completed within the 400-step limit. At the same time, it is evident that the performance among the five policies is quite similar, indirectly indicating the stability of the learning process and outcomes. More detailed results are summarized in Table 6 and Table 7.

In conclusion, the results above confirm that under constant-speed control, PP control is insufficient to handle diverse path scenarios. However, the outcomes of our proposed method demonstrate that without a path-specific designed reference velocity, an adaptive velocity control strategy is learned and greatly enhances path following performance under the two criteria we examined.

#### 4.2.2. Path Convergence and Adaptive Velocity

The test path is an eight-shaped curve that is widely used for testing path following algorithm, as defined in Equation (26). It includes straight segments as well as curves with varying degrees of curvature, representing commonly encountered scenarios in real-world applications.

(26) $$\left\{\begin{array}{cc} x & =a\sin(\lambda )\\ y & =a\sin(\lambda )\cos(\lambda ) \end{array}\right.$$

where *a* is a constant that determines the size and shape of the curve, set as 1.0. Notably, despite the provided parameterization equation for the path here, it is still necessary to undergo arc-length parameterization, as referenced in [38]. Moreover, based on the algorithm for generating random paths and multiple tests, as examined in Figure 8, this specific path is highly unlikely to occur in the training environment.

**Figure 10:**
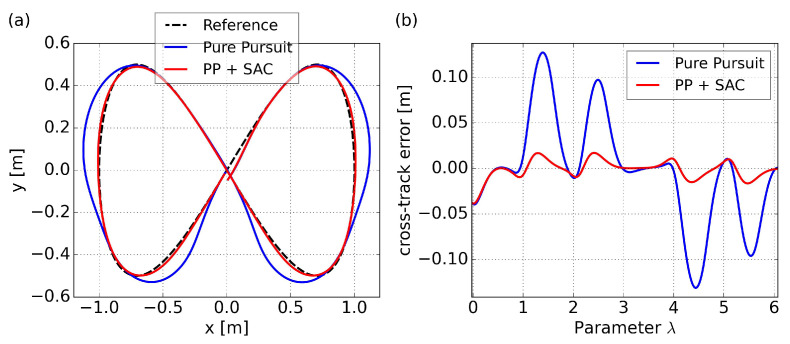
Path following comparison of the eight-shaped path in simulation: (**a**) trajectories results; (**b**) cross-track error results.

**Figure 11:**
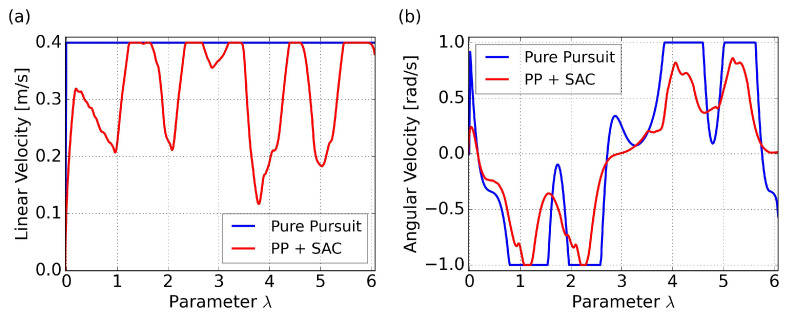
Velocity comparison for the eight-shaped path in simulation: (**a**) linear velocity results; (**b**) angular velocity results.

The trajectory and cross-track error results for tracking the eight-shaped curve using PP control and the proposed SAC-based control are illustrated in Figure 10, with velocity comparisons presented in Figure 11. As a comparison, the results of the PP control are based on a velocity command of 0.4 m/s. Since our proposed method aims to maximize velocity, and considering that we can view this specific path as a kind of randomly generated trajectory, we are uncertain about how to set a reference velocity for PP control in this particular scenario. The initial posture is set to a randomly generated value of [0.009,−0.044,0.736] for both methods.

It is noteworthy that, due to the differing time consumption of the two methods, utilizing time as the horizontal axis for comparing cross-track errors and velocity changes may not yield a clear comparison. The use of λ as the horizontal axis allows for a more accurate comparison of the two methods at the same path curvature. Subsequent graphical comparisons will follow the same principle.

**Figure 12:**
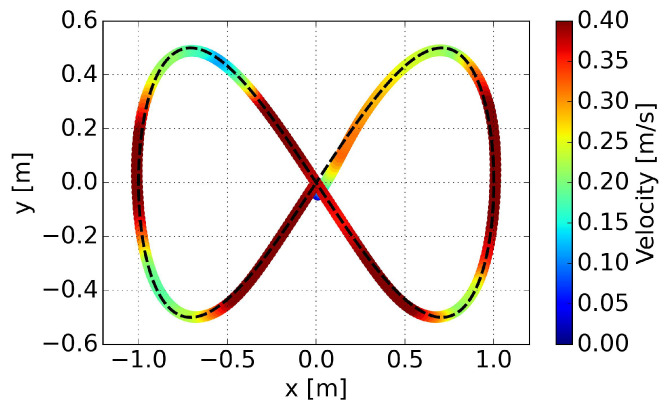
Linear velocities along the trajectory for the eight-shaped path in simulation.

The results of the PP control indicates poor performance in curved sections, due to excessively high velocity and saturated angular velocities, resulting in insufficient turning. On the other hand, our proposed method, specifically policy 4 in the figure, can achieve minimal cross-track error due to adaptive velocity adjustment. This significantly improves the success rate of path following and prevents entering the saturation area of angular velocity. Specific comparative results are summarized in Table 8, where $\bar{e_{p}}$ represents the root mean squared cross-track error, ${|e_{p}|}_{max}$ represents the maximum absolute error occurred, and $\bar{v}$ represents the average velocity. Both path convergence and velocity performance are consistently demonstrated across the five trained policies. A more intuitive visualization is presented in a trajectory scatter plot plotted using velocity magnitude, as shown in Figure 12.

It is evident that the proposed method can adjust velocity in a smooth way, decelerating before entering a curve, accelerating when exiting a curve rapidly, and maintaining maximum velocity on relatively straight segments. Smooth variations in linear velocity are also evident in this representation.

### 4.3. Experimental Results

#### 4.3.1. Experimental Setup

**Figure 13:**
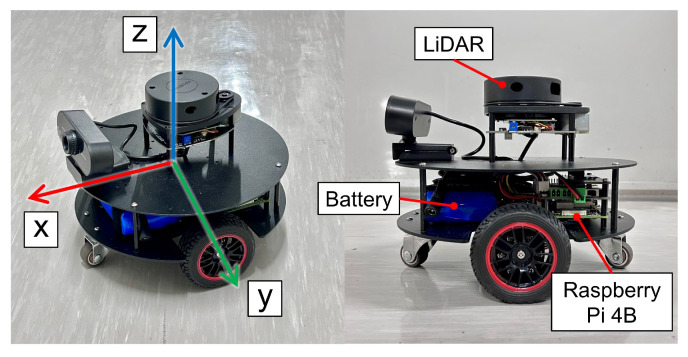
Configuration of the experimental nonholonomic wheeled mobile robot.

The experimental nonholonomic wheeled mobile robot, as depicted in Figure 13, features the placement of the active wheel at the center of the chassis, with an additional omnidirectional wheel at both the front and rear. The wheelbase, measured and calibrated to 0.172 m, corresponds precisely to the parameter employed in simulation. The overall dimensions of the robot measure 216×216×171 mm.

At the top layer of the robot, a 360 Degree Laser Scanner, utilizing Laser imaging, Detection, and Ranging (LiDAR), is configured for subsequent mapping and localization functions. This setup serves the purpose of perceiving the robot’s posture. The middle layer comprises the main control Raspberry Pi 4 Model B, motor control module, and battery. Although an RGB camera is also configured, it was not utilized in this paper.

**Figure 14:**
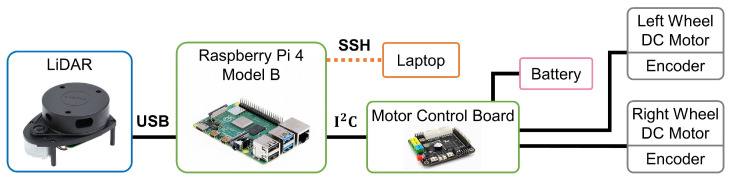
Diagram of the robot’s connectivity and communication.

The Raspberry Pi operates on the Ubuntu 20.04 system to support the execution of the Robot Operating System (ROS), a set of software libraries and tools designed to facilitate the development of robot applications. This content specifically utilizes the ROS Noetic version. Figure 14 illustrates the robot’s connectivity and communication. This necessitates that the laptop and the Raspberry Pi system be within the same local area network for effective communication, with the ROS facilitating the connection and interaction.

The primary focus of this paper is path following; therefore, the methods for obtaining the real-world posture of the robot are briefly summarized. Given that a robot equipped with LiDAR has already been configured, the experiments are conducted indoors. The mapping component utilizes Gmapping [39], a Simultaneous Localization and Mapping (SLAM) algorithm based on 2D laser range data for constructing 2D grid maps. After obtaining the map, during the execution of the path following algorithm, the robot’s posture is determined using Adaptive Monte Carlo Localization (AMCL) [40], which employs a particle filter to track the robot’s posture against a known map.

Due to the fact that the policy for velocity control is trained using PyTorch modules, in order to use it on the Raspberry Pi, we have converted it to the Open Neural Network Exchange (ONNX) format. In one control cycle, the measured and computed observations, obtained through the robot’s posture via AMCL, are input into the ONNX model. The resulting velocity commands are then published at a frequency of 20 Hz. Table 9 summarizes the parameter count, Floating-Point Operations (FLOPs), and the inference time during actual operation of our model. The results highlight the remarkable lightweight nature and computational efficiency of our model, perfectly fulfilling our control objectives.

#### 4.3.2. Path Convergence and Adaptive Velocity

The trained policies were found to be capable of tracking paths with small cross-track errors in experimental testing, similar to simulation results. However, the actual velocity was observed to be lower in reality compared to simulation. We attribute this difference in velocity tracking to delays in input–output caused by communication issues inherent in real-world robots. This phenomenon has also been identified and discussed in [41,42]. In this paper, we employ the method of modifying the control dynamics of the robot by scaling the output of the policy as used in [42] for its simplicity. After testing, an appropriate scaling factor in experiment was determined to be 2.2.

**Figure 15:**
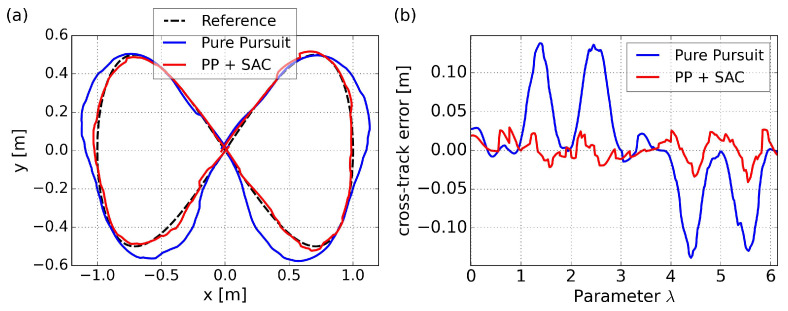
Path following comparison of the eight-shaped path in experiment: (**a**) trajectories results; (**b**) cross-track error results.

**Figure 16:**
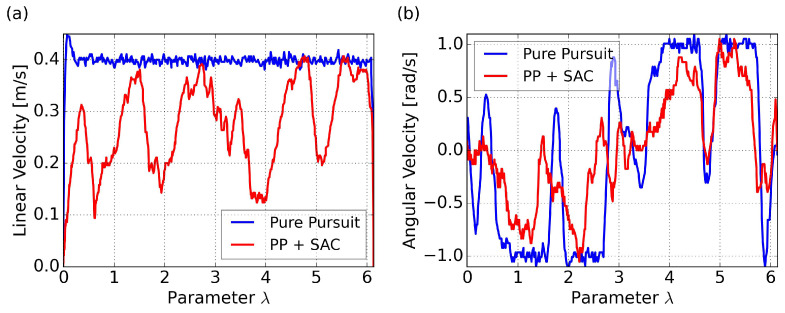
Velocity comparison for the eight-shaped path in experiment: (**a**) linear velocity results; (**b**) angular velocity results.

The trajectory and cross-track error results for tracking the eight-shaped path are shown in Figure 15, demonstrating consistent results with the simulation. Specifically, the results in the figure represent the outcomes of policy 2. The acceleration of the policy in the experiment appears more cautious, attributed to the transfer relationship and delay between input and output of motor wheels. Setting aside this aspect, the consistency of the policies has been validated, and a noticeable deceleration is observed in the curved segments. Similarly, the proposed method’s angular velocity avoids the shortcomings of PP control, preventing steering from entering the saturation region. The results of the five experiments are summarized in Table 10. The experimental results for the five policies consistently outperform traditional PP control in both RMSE and maximum error. However, due to the slower velocity on straight segments in the middle portion compared to the simulated speed (see Figure 16), there is a slight overall decrease in average velocity. Video S1, which is an experimental recording captured from a bird’s-eye view perspective, can be found in the supplementary materials for reference.

**Figure 17:**
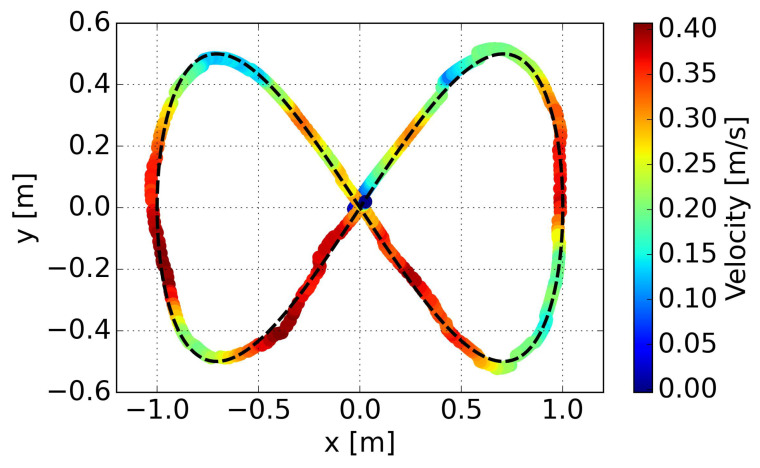
Linear velocities along the trajectory for the eight-shaped path in experiment.

Velocities along the trajectory in the experiment are visualized in Figure 17. Similar performance to the simulation is confirmed, with deceleration observed before entering a curve and rapid acceleration when exiting the curve. This behavior ensures reduction in cross-track error and success in steering.

## 5. Conclusions

In this paper, we propose a path-following control method that combines traditional steering control with DRL. Through interactive learning in a stochastic environment, the proposed method is demonstrated to have learned an adaptive velocity control strategy capable of addressing various path scenarios. It is quantitatively evaluated using two criteria: failure rate and completion rate. The results show a significant out-performance in both criteria compared to constant speed control under PP control. Notably, our method demonstrates robustness across diverse paths without the need for repeated design of reference velocities. Both simulation and experimental tests on an eight-shaped path confirm the learned control strategy’s ability to reduce cross-track error and achieve smooth velocity adjustments. This is manifested by deceleration before entering a curve, rapid acceleration when exiting a curve, and maintaining maximum velocity on relatively straight segments. The entire approach underscores the powerful capabilities of DRL in addressing path-following challenges.

Despite the achievements we have made, we discovered that directly applying the trained policies in experiments, while successful in path-following tasks with minimal cross-track error, exhibits differences in velocity tracking compared to simulation. This discrepancy is primarily attributed to the additional uncertainties in the control dynamics of the real-world robot, including input–output delays caused by communication. Recent research has delved into RL in delayed environments, suggesting the potential to enhance velocity tracking performance and bridge the gap between simulation and the real world [41]. On the other hand, safety considerations, which are often crucial in practical robot scenarios, were not taken into account when formulating the problem. Exceeding a certain threshold of cross-track error may be deemed unsafe. Incorporating control barrier functions [43] into the problem has been proven as an effective and successful approach when safety control becomes a necessary consideration [44,45]. We will address the aforementioned shortcomings in future work.

In conclusion, our proposal contributes to the advancement of autonomous mobility by integrating conventional algorithm with state-of-the-art DRL techniques, thereby enhancing the robustness of path following. Moreover, we believe its potential for broader application to vehicles constrained by nonholonomic principles, extending beyond the specific model studied in this paper. 

## Figures and Tables

**Table 1:** Mobile robot parameters.

Symbol	Description	Value
$\hat{v}$	Set maximum linear velocity	0.4 m/s
$\hat{\omega }$	Set maximum angular velocity	1.0 rad/s
*b*	Wheelbase	0.172 m

**Table 2:** Hyperparameters in training the SAC for path following.

Description	Value
Sampling period	0.05 s
Target soft update rate	0.005
Discount factor	0.99
Entropy target	−1
Maximum time steps per episode	$4\times 10^{2}$
Warm-up time steps	$5\times 10^{3}$
Maximum time steps	$5\times 10^{5}$
Experience replay buffer size	$5\times 10^{5}$

**Table 3:** Hyperparameters of both actor and critic networks for path following.

Description	Value
Number of hidden layers	2
Number of neurons per layers	256
Activation function	ReLU
Optimizer	Adam
Learning rate	$3\times 10^{-4}$
Minibatch size	256

**Table 4:** Failure rates of the PP control at various reference velocities for three cross-track error thresholds.

Velocity [m/s]	Threshold [m]		
	0.1	0.2	0.3
0.10	0.000	0.000	0.000
0.15	0.075	0.000	0.000
0.20	0.276	0.053	0.002
0.25	0.486	0.257	0.043
0.30	0.606	0.431	0.238
0.35	0.698	0.562	0.394
0.40	0.767	0.643	0.516

**Table 5:** Completion rates of the PP control at various reference velocities for three cross-track error thresholds. For instance, the value 0.400±0.077 indicates the mean result with a standard deviation range, derived from 1000 tasks.

Velocity [m/s]	Threshold [m]		
	0.1	0.2	0.3
0.10	0.400±0.077	0.400±0.077	0.400±0.077
0.15	0.578±0.132	0.597±0.112	0.597±0.112
0.20	0.678±0.215	0.758±0.150	0.776±0.122
0.25	0.699±0.279	0.803±0.234	0.893±0.130
0.30	0.671±0.312	0.773±0.287	0.865±0.237
0.35	0.619±0.314	0.710±0.307	0.802±0.281
0.40	0.571±0.307	0.662±0.312	0.739±0.300

**Table 6:** Failure rates of proposed SAC-based path following control for three cross-track error thresholds.

Method	Threshold [m]		
	0.1	0.2	0.3
Policy 1	0.155	0.004	0.000
Policy 2	0.114	0.005	0.000
Policy 3	0.157	0.005	0.000
Policy 4	0.118	0.005	0.000
Policy 5	0.262	0.008	0.000

**Table 7:** Completion rates of proposed SAC-based path following control for three cross-track error thresholds. For instance, the value 0.883±0.284 indicates the mean result with a standard deviation range, derived from 1000 tasks.

Method	Threshold [m]		
	0.1	0.2	0.3
Policy 1	0.883±0.284	0.984±0.070	0.987±0.070
Policy 2	0.891±0.285	0.972±0.124	0.975±0.124
Policy 3	0.880±0.286	0.981±0.080	0.985±0.080
Policy 4	0.892±0.277	0.975±0.099	0.978±0.099
Policy 5	0.817±0.326	0.968±0.140	0.971±0.140

**Table 8:** Results for one lap of the eight-shaped path in simulation.

Method	${\bar{e}}_{p}$ [m]	${|e_{p}|}_{\max}$ [m]	$\bar{v}$ [m/s]
Pure Pursuit	0.0593	0.1311	0.4000
Policy 1	0.0117	0.0385	0.2868
Policy 2	0.0118	0.0385	0.2793
Policy 3	0.0119	0.0384	0.2819
Policy 4	0.0115	0.0385	0.2688
Policy 5	0.0121	0.0385	0.2958

**Table 9:** Computational cost in real-time, where the inference time is an average of 100 inferences.

Parameters	FLOPs	Inference Time
67,842	67,328	97.8 μs

**Table 10:** Results for one lap of the eight-shaped path in experiment.

Method	${\bar{e}}_{p}$ [m]	${|e_{p}|}_{\max}$ [m]	$\bar{v}$ [m/s]
Pure Pursuit	0.0674	0.1386	0.3949
Policy 1	0.0207	0.0493	0.2317
Policy 2	0.0146	0.0410	0.2257
Policy 3	0.0108	0.0314	0.2444
Policy 4	0.0134	0.0376	0.2097
Policy 5	0.0117	0.0368	0.2090

## Data Availability

The data that support the findings of this study are available from the author, Seiji Hashimoto, upon reasonable request.

## Supplementary Materials

The following supporting information can be downloaded at: www.mdpi.com/xxx/s1, Video S1: Path following comparison of the eight-shaped path in experiment.

## Abbreviations

The following abbreviations are used in this manuscript:

AMR	Autonomous Mobile Robot
PID	Proportional-Integral-Derivative
PP	Pure Pursuit
RL	Reinforcement Learning
DNN	Deep Neural Network
DRL	Deep Reinforcement Learning
SAC	Soft Actor-Critic
MDP	Markov Decision Process
MERL	Maximum Entropy Reinforcement Learning
TD	Temporal-Difference
ReLU	Rectified Linear Unit
LiDAR	Laser imaging, Detection, and Ranging
ROS	Robot Operating System
SLAM	Simultaneous Localization and Mapping
AMCL	Adaptive Monte Carlo Localization
ONNX	Open Neural Network Exchange
FLOP	Floating-Point Operation
